# Searching for New Gold(I)-Based Complexes as Anticancer and/or Antiviral Agents

**DOI:** 10.3390/molecules30081726

**Published:** 2025-04-11

**Authors:** Paola Checconi, Annaluisa Mariconda, Alessia Catalano, Jessica Ceramella, Michele Pellegrino, Stefano Aquaro, Maria Stefania Sinicropi, Pasquale Longo

**Affiliations:** 1 Department for the Promotion of Human Sciences and Quality of Life, San Raffaele University, Via di Val Cannuta 247, 00166 Rome, Italy; paola.checconi@uniroma5.it; 2 Laboratory of Microbiology, IRCCS San Raffaele Roma, Via di Val Cannuta 247, 00166 Rome, Italy; 3 Department of Basic and Applied Sciences, University of Basilicata, Via dell’Ateneo Lucano, 10, 85100 Potenza, Italy; annaluisa.mariconda@unibas.it; 4 Department of Pharmacy-Drug Sciences, University of Bari “Aldo Moro”, Via Orabona, 4, 70126 Bari, Italy; 5 Department of Pharmacy, Health and Nutritional Sciences, University of Calabria, Via Pietro Bucci, 87036 Arcavacata di Rende, Italy; jessica.ceramella@unical.it (J.C.); michele.pellegrino@unical.it (M.P.); s.sinicropi@unical.it (M.S.S.); 6 Department of Life, Health and Environmental Sciences, University of L’Aquila, Piazzale Salvatore Tommasi, 1, Blocco 11, 67010 L’Aquila, Italy; stefano.aquaro@univaq.it; 7 Department of Chemistry and Biology “A. Zambelli”, University of Salerno, Via Giovanni Paolo II, 132, 84084 Fisciano, Italy; plongo@unisa.it

**Keywords:** gold–NHC, antiviral, anticancer, *N*-heterocyclic carbenes, auranofin

## Abstract

Approaches capable of simultaneously treating cancer and protecting susceptible patients from lethal infections are highly desirable, although they prove challenging. Taking inspiration from the well-known anticancer platinum complexes, successive studies about the complexation of organic compounds with other late transition metals, such as silver, gold, palladium, rhodium, ruthenium, iridium, and osmium, have led to remarkable anticancer activities. Among the numerous chemical moieties studied, *N*-heterocyclic carbenes (NHCs) have revealed very attractive activities due to their favorable chemical properties. Specifically, gold–NHC complexes emerged as some of the most active complexes acting as antitumor agents. On the other hand, some recent studies have highlighted the involvement of these complexes in antiviral research as well. The well-known gold-based, orally available complex auranofin approved by the Food and Drug Administration (FDA) for the treatment of rheumatoid arthritis has been suggested as a repositioned drug for both cancer and viral infections. In the era of the COVID-19 pandemic, the most interesting goal could be the discovery of gold–NHC complexes as dual antiviral and anticancer agents. In this review, the most recent studies regarding the anticancer and antiviral activities of gold(I)–NHC complexes will be analyzed and discussed, offering an interesting insight into the research in this field.

## 1. Introduction

**Figure 1:**
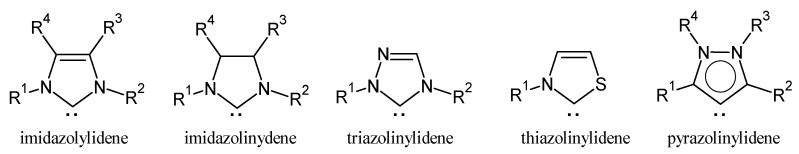
Structures of NHCs.

Lethal viral infections and co-infections, such as those caused by SARS-CoV-2, HIV, monkeypox, and influenza viruses, have spread worldwide. Concurrently, the number of cancer patients and cancer deaths is still continuously increasing. Hence, numerous research is addressed to find new drugs acting both as antitumor and antiviral agents, and amazing results have been obtained with metal complexes [1,2,3,4,5,6]. Among these, metal *N*-heterocyclic carbene (NHC) complexes (Figure 1) have represented a wonderful discovery as they present entirely appropriate prerequisites for effective drug design and quick optimization, and numerous scientific studies are being carried on in this view [6].

**Figure 2:**
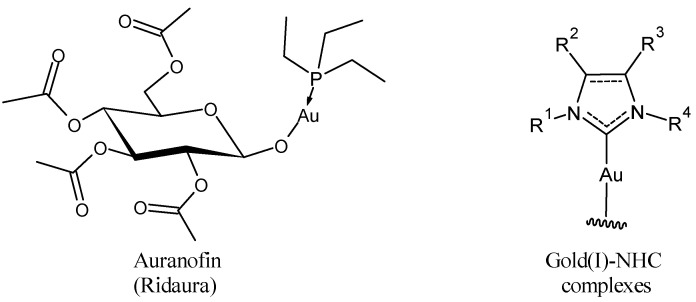
Structure of gold complexes.

Advances in the study of the antitumor properties of metal–NHC complexes, including Au, Ag, Pd, Pt, Ru, Rh, Re, Ir, Fe, and Cu, have recently been reviewed by Zhang et al. [7]. However, except for well-known platinum derivatives, none of the metallodrugs received approval for clinical chemotherapy, even though numerous candidates have entered clinical trials, such as Ru(III) complexes NAMI-A and BOLD-100, Ru(II) RAPTA-C and TLD1433 [8], Cu(II) complex Elesclomol [9], the Casiopeinas family, mixed chelate copper complexes, and the Au(I) complex Auranofin (Figure 2). The topical Co(III) chelate CTC-96 has been demonstrated to reduce the severity of conjunctival viral infection and make its resolution faster in the in vivo model of keratoconjunctivitis consisting of rabbits infected with the Adenovirus type 5 (ad-5) and to have virucidal and antiviral activity against ad-5-infected HeLa, A549, and SIRC cell lines [10].

Specifically, the gold complex auranofin (Ridaura) received its first approval by the FDA in 1985 as an antiarthritic agent [11]. Then, based on several preclinical studies [12], it was suggested as a repositioning drug behaving as an anticancer, antimicrobial, antiviral, and antiparasitic agent [13,14,15,16]. Among the numerous gold-based complexes [17,18], gold–NHC complexes are widely studied for their very interesting anticancer activities [19,20,21,22,23,24], as well as silver–NHC complexes [25]. Numerous molecular modeling studies on gold– and silver–NHC as anticancer complexes continue to be proposed, in order to find new compounds [26,27,28,29,30,31] and/or to better understand their mechanism of action [32]. In addition, recent reports have described the involvement of gold– and silver–NHC complexes [33,34,35] in the antiviral activity against SARS-CoV-2 by inhibiting SARS-CoV-2 papain-like protease PL^pro^, which is a key enzyme in the virus replication in infected cells. In this review, we focused our attention on auranofin and gold(I)–NHC complexes and summarized the most interesting studies on these complexes with antiviral and/or anticancer activities carried out in recent years, including their sources, features, and mechanisms of action.

## 2. Auranofin: The First Gold Complex in Clinics

**Figure 3:**
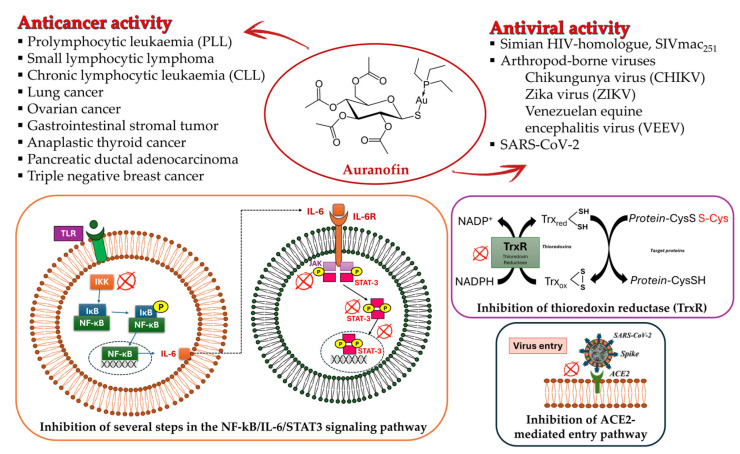
Anticancer and antiviral activities of Auranofin.

Auranofin (1-thio-β-D-glucopyranosatotriethylphosphine gold-2,3,4,6-tetraacetate) is an orally available lipophilic (LogP = 1.6 [36]) gold(I) complex used in therapy. Following its approval as an anti-inflammatory agent for rheumatoid arthritis, it was investigated for other potential therapeutic applications in a number of diseases, including cancer, viral, parasitic, and bacterial infections (Figure 3) [37,38,39,40,41]. Auranofin has also been studied in vitro and in vivo in nonalcoholic fatty liver disease [42] and for neurodegenerative diseases such as Alzheimer’s disease and multiple sclerosis [43]. Different studies have been carried out regarding the anticancer activity in prolymphocytic leukemia (PLL)/small lymphocytic lymphoma/chronic lymphocytic leukemia (CLL) [12,44], lung cancer [45,46,47], ovarian cancer [48], gastrointestinal stromal tumor [49], and recently in anaplastic thyroid cancer [50]. It completed phase 1 and 2 clinical trials as anticancer in chronic lymphocytic leukemia (NCT01419691), ovarian and lung cancer (NCT01737502, NCT01747798, NCT03456700) [51,52] and has recently been suggested for the treatment of pancreatic ductal adenocarcinoma [53] and triple-negative breast cancer [54]. Studies related to the outcomes of the combination of auranofin with radiation therapy are controversial. Nag et al. (2019) [55] reported that auranofin pretreatment was able to prevent radiation toxicity in colon tumor in studies in organoids, whereas Lee et al. (2019) [56] demonstrated that it aggravates radiation-induced acute intestinal injury in mice.

Auranofin was one of the first metallodrugs showing potential antiviral activity. The first report was about an HIV-positive and psoriatic arthritis patient, under treatment with auranofin, who showed a significant, sustained increase in CD4 T-cells, the main virus cell target, and resistance to opportunistic infections [57]. Later, it was demonstrated that auranofin led to a reduction in the viral reservoir in peripheral blood cells from antiretroviral therapy (ART)-treated Chinese rhesus macaques infected with the simian HIV homolog SIVmac_251_; moreover, macaques that received auranofin showed a deferred and blunted viral load rebound after ART suspension [58]. In the clinical trial NCT02961829, auranofin was well tolerated and decreased total viral and integrated proviral DNA, in patients under intensified ART [59], with higher activity than chloroquine [60]. The efforts of the new combined therapies, in fact, try to target all the HIV cellular reservoirs, critical factors to viral eradication, since they harbor nonproductive provirus, which reactivates viral replication and leads to viremia rebound when ART is suspended [58,61]. Auranofin has been tested against some arthropod-borne viruses that have re-emerged in recent decades, causing large-scale epidemics in many parts of the world. Langsjoenm et al. (2017) [62] reported that auranofin was able to inhibit the Chikungunya virus (CHIKV) replication in vitro and reduced viremia and morbidity in a murine model of infection. Moreover, it inhibited flavivirus Zika virus (ZIKV) and alphavirus Venezuelan equine encephalitis virus (VEEV) replication in vitro. All three of these viruses are enveloped RNA viruses in which glycoproteins required oxidative folding pathways that the authors suggested could represent auranofin targets [62]. During the COVID-19 pandemic [63,64], auranofin was suggested as a repositioned drug acting against SARS-CoV-2 [65,66,67]. Rothan et al. (2020) [68] demonstrated that the treatment of hepatocarcinoma cells infected with SARS-CoV-2 with auranofin reduced viral RNA copies and the expression of inflammatory cytokines, at a low micromolar concentration (EC_50_ = 1.4 μmol/L), suggesting that it could be a useful drug to limit both viral replication and associated inflammatory injury. Different pathways have been suggested to explain auranofin mechanisms of action in different diseases. The anti-inflammatory activity in rheumatoid arthritis is likely related to the inhibition of thioredoxin reductase (TrxR) and thioredoxin glutathione reductase (TGR) enzymes, thus leading to control reactive oxygen species (ROS) and DNA damage. Moreover, the inhibition of TrxR prevents NF-kB-DNA binding and gene expression. In addition, it has been suggested that it inhibits several steps in the NF-kB/IL-6/STAT3 inflammatory signaling pathway [66]. Auranofin has been shown in fact to suppress TLR4 homodimerization [69]. Jeon et al. (2000) [70] reported that it prevents the activation of IkB kinase (IKK) that promotes the phosphorylation of IkB and translocation of NF-kB in the nucleus and its activity [14,15]. Moreover, it acts also directly on inhibitory IkB proteins associated with NF-kB by preventing their degradation and therefore holding NF-kB in the cytosol. Auranofin inhibits the IL-6-induced phosphorylation of Janus kinase 1 (JAK1) and signal transducer and activator of transcription 3 (STAT3). The inhibitory effect of auranofin on STAT3 translocation to the nucleus also leads to the blockage of STAT3-regulated gene expression [71]. The anti-inflammatory effect of auranofin was studied by Hwangbo et al. (2021) [38] in a model of chronic inflammation represented by palmitic acid and a low concentration of lipopolysaccharide (LPS)-stimulated macrophages, demonstrating the interaction of the drug with TLR4 and downregulation of NADPH oxidase (NOX)4-mediated NF-κB signaling pathway. It was shown to modulate TNF-α, IL-8, and IL-6 secretion by macrophages and monocytes.

The anticancer activity of auranofin has been suggested to occur through the inhibition of redox enzymes that are essential for maintaining intracellular levels of ROS, interaction with TrxR, ubiquitin–proteasome system (UPS), tyrosine kinases, protein tyrosine phosphatases (PTPs), topoisomerases, and signaling pathways related to cell proliferation, apoptosis, and ferroptosis [72,73,74,75]. The NF-kB/IL-6/STAT3 cascade may also be useful to explain the anticancer activity of auranofin, as NF-kB, IL-6, and STAT3 are central players linking chronic inflammation to cancer by driving tumor initiation and subsequent growth and metastasis.

The mechanism of antiviral activity could be explained again considering the inhibition of redox enzymes and related pathways, which play a key role in viral infections [76]. For instance, TrxR is important for the redox conformational changes that HIV glycoprotein gp120 undergoes, allowing the virus to enter cells [77]. Regarding coronaviruses, multiple mechanisms have been proposed. Laplantine et al. (2022) [78] also showed a dual inhibitory effect of auranofin on SARS-CoV-2 infection: it inhibited the ACE2-mediated entry pathway and dampened virus-induced inflammation by preventing post-translational modifications of NF-kB effectors and their recruitment into activating complexes. Since high levels of IL-6 are linked with COVID-19 severity and mortality, the auranofin effect on NF-kB/IL-6/STAT3 cascade could be an advantage in the management of the infection [66]. Furthermore, Gil-Moles and colleagues (2020) [34] reported that auranofin inhibited both SARS-CoV-2 spike–ACE2 interactions and papain-like protein (PL^pro^) (IC_50_ = 25.5 ± 1.2 μM and IC_50_ = 0.75 ± 0.13 μM against SARS-CoV PL^pro^ and SARS-CoV-2 PL^pro,^ respectively). In a more recent study, the same research group [79] showed that the mechanism of SARS-CoV-2 PL^pro^ inhibition by other metallodrugs, with silver, involves the ejection of the zinc(II) ion from the zinc binding domain of the viral protease, in agreement with previous studies of the Abbehausen research group that had already shown HIVNCp7 zinc finger inhibition by gold(I)-phosphine-*N*-heterocycles [80]. Ott and co-workers [33,66], on the other hand, screened a great selection of coordination chemotypes bearing a variety of metal ions, and ligands against PL^pro^, in which the Au(I) compounds presented striking results. The best candidates were aurothiomalate, [AuCl(Et_3_P)], and other Au(I)–NHCs bearing the structure AuCl(NHC).

## 3. Gold(I)–NHC Complexes

Starting from auranofin as a lead compound, several gold(I) complexes have been studied for their various activities, also behaving as dual agents against two diseases, including cancer and viral infections. Given the usefulness of NHCs for biological activities, several metal–NHC complexes have been studied that behave as dual agents, such as silver(I)–NHC and ruthenium(II)–NHC complexes, which act as anticancer molecules and antibacterials [81], or presenting more than two activities [82].

Gold(I)–NHC complexes emerged for their interesting properties in organic [83] and inorganic chemistry [84,85,86] and their biological activities [87], such as anticancer, antiviral, antioxidant, anti-inflammatory, and antimicrobial activities, also against antibiotic resistant bacterial pathogens [88,89,90,91].

### 3.1. Gold–NHC Complexes as Anticancer Agents

Several studies aim to find new gold–NHC complexes acting as anticancer molecules. Different targets have been suggested to explain the mechanism of action of these compounds [20], resembling those suggested for auranofin, including TrxR inhibition [92,93,94,95,96,97,98] interaction with UPS, tyrosine kinases, PTPs [99], and topoisomerases [21].

As shown in Table 1, we summarized some recent studies of cytotoxicity, and the half-maximal (50%) inhibitory concentration (IC_50_) values are given. The MTT (3-(4,5-dimethylthiazol-2-yl)-2,5-diphenyltetrazolium bromide) cell viability test was adopted to evaluate the cytotoxic effects of the complexes, unless otherwise indicated. The standards used and IC_50_ values against non-tumoral cells are detailed in the text.

Trávníček et al. (2024) [100] studied a series of NHC–gold(I) complexes, involving 1,3-bis(2,6-diisopropylphenyl)imidazol-2-ylidene ligand in combination with 6-mercaptopurine derivatives, and evaluated the in vitro cytotoxicity against four human cancer cell lines: A2780 (ovarian) and A2780R (ovarian cisplatin resistant), PC3 (prostate), and MCF-7 (breast). Normal human MRC-5 cells (lung fibroblasts) were used for selectivity. The standard was cisplatin (IC_50_ = 17.2 ± 0.9, > 50, > 50 and 25.3 ± 3.6 µM against A270, A270R, PC3, and MCF-7, respectively). Complexes **1**–**3** showed significative cytotoxicity against all cell lines, with the highest IC_50_ values ranging between 3.4 and 6.2 μM against A2780 cell lines, also demonstrating a strong pro-apoptotic effect and the loss of mitochondrial membrane potential, as shown by flow cytometry assays. Thus, the authors suggested the main mechanism of action based on the collapse of mitochondrial metabolism and the activation of the intrinsic signaling pathway of apoptosis, resulting in cell death. The complexes also showed a moderate selectivity index (SI) and resistance factor RF [IC_50_(A2780R)/IC_50_(A2780)].

D’Amato et al. (2024) [101] studied a series of hybrid molecules in which NHC-Au(I) complexes and *N*-alkylthiolated carbazoles are linked together and evaluated their anticancer activity against breast cancer cells, MCF-7 and MDA-MB-231. The best anticancer activity was obtained for complexes **4**–**6**, all of them being more potent than the reference cisplatin (IC_50_ = 34.3 ± 1.0 and 27.3 ± 1.1 μM against MCF-7 and MDA-MB-231 cells, respectively). The SI was calculated in comparison to normal human mammary epithelial cell line MCF-10A, and the three complexes were found to be highly selective.

Marra et al. (2024) [102] described some chiral gold(I) and silver(I) NHC complexes, evidencing the influence of chirality on anticancer activity. Complex **(*R*)-7** was more active than its enantiomer against both MCF-7 and MDA-MB-231 breast cancer cell lines, whereas **(*R*)-8** showed a strange behavior, being more active than its enantiomer only against MDA-MB-231 cells. IC_50_ against normal MCF-10A cell lines was also different depending on the enantiomer tested (IC_50_ = 52.9 ± 2.2 and 64.5 ± 3.2 μM for **(*R*)-** and **(*S*)-8**, respectively, and IC_50_ = 38.0 ± 2.2 and 17.2 ± 1.6 μM for **(*R*)-** and **(*S*)-7,** respectively). The highest selectivity was found for **(*R*)-7**.

Al-Buthabhak et al. (2024) [103] reported a study on a series of gold–NHC complexes with a thiocarboxylate ligand against cisplatin-resistant ovarian cancer cells (OVCAR-8) that showed promising anticancer activity, with IC_50_ values lower than 10 μM. The most potent were **9**–**11**. Positive and negative controls were gold–NHC complexes, as previously reported [104].

Ma et al. (2024) [105] reported a series of dinuclear gold–-NHC–thiolate complexes with remarkable cytotoxicity towards several cancer cell lines, namely cisplatin-sensitive ovarian cancer cells (A2780), cisplatin-resistant ovarian cancer cells (A2780cis), colon cancer cells (HCT116), lung cancer cells (A549), and triple-negative breast cancer (MDA-MB231). Complexes **12**–**14** showed remarkable cytotoxicity, being more active than cisplatin against all cell lines (cisplatin, IC_50_ = 1.1 ± 0.1, 11 ± 3, 21 ± 7, 9 ± 1 and 15 ± 1 μM A2780, A2780cis, HCT116, A549, and MDA-MB231, respectively).

Galassi et al. (2024) [106] reported an in-depth study of gold(I/III)–NHC complexes on non-small-cell lung cancer (NSCLC) cell lines, with or without KRAS mutations (A549, H460, H1792, HCC-44, H1355, and H522, H661, H1395, H1993, respectively) to study the mechanism of action of the synthesized complexes. Gold(I)–NHC complex **15** exerted the highest cytotoxicity (average IC_50_ values are given in the table) and a high degree of TrxR inhibition in both H522 and A549 cell lines after 24 h of treatment. Moreover, it reduced the enzymatic activity of hDHFR by 80% at a dihydrofolate concentration comparable with Km, with an IC_50_ of about 15 mM. Thus, the authors confirmed the importance of TrxR in the mechanism of action of these complexes and suggested a dual-target inhibition of both human DHFR and TrxR enzymes for this complex.

Mariconda et al. (2024) [107] reported a study on silver and gold complexes with NHC derived from caffeine. Complex **16** showed anticancer activity against breast cancer MDA-MB-231 and MCF-7 cell lines, being even more active than cisplatin (IC_50_ = 32.2 ± 1 µM against MDA-MB-231; IC_50_ = 26.2 ± 1 µM against MCF-7) against MDA-MB-231 cells. The compound was not cytotoxic against normal cells (MCF-10A), showing IC_50_ > 100 µM.

**Table 1:** Au–NHC complexes as anticancer agents.

Structure	Compound	Anticancer Activity	Reference
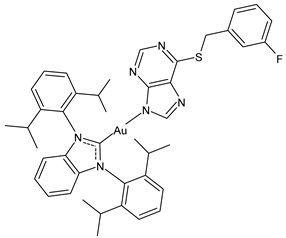	**1**	IC_50_ = 3.4 ± 0.4 µM (A270) IC_50_ = 6.9 ± 1.7 µM (A270R) IC_50_ = 11.3 ± 0.1 µM (PC3) IC_50_ = 15.7 ± 1.1 µM (MCF-7)	[100]
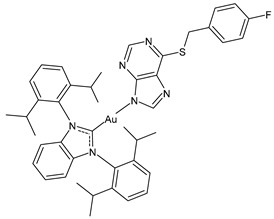	**2**	IC_50_ = 3.5 ± 0.3 µM (A270) IC_50_ = 7.3 ± 2.1 µM (A270R) IC_50_ = 11.2 ± 0.6 µM (PC3) IC_50_ = 14.9 ± 1.2 µM (MCF-7)	[100]
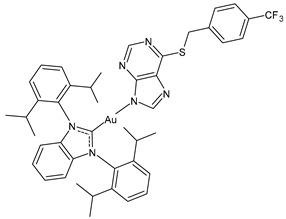	**3**	IC_50_ = 6.2 ± 0.7 µM (A270) IC_50_ = 12.1 ± 0.7 µM (A270R) IC_50_ = 15.5 ± 2.3 µM (PC3) IC_50_ = 27.3 ± 1.2 µM (MCF-7)	[100]
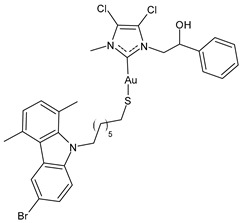	**4**	IC_50_ = 3.6 ± 0.5 μM (MCF-7) IC_50_ = 6.4 ± 0.4 μM (MDA-MB-231)	[101]
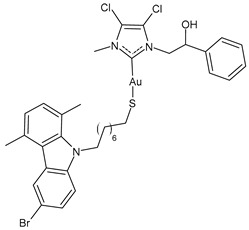	**5**	IC_50_ = 3.5 ± 0.2 μM (MCF-7) IC_50_ = 6.3 ± 0.7 μM (MDA-MB-231)	[101]
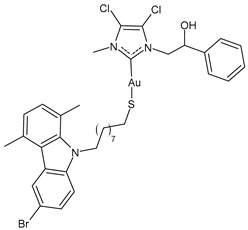	**6**	IC_50_ = 2.7 ± 0.3 μM (MCF-7) IC_50_ = 6.1 ± 0.2 μM (MDA-MB-231)	[101]
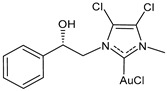	**(*R*)-7**	IC_50_ = 2.2 ± 0.2 μM (MCF-7) IC_50_ = 1.2 ± 0.2 μM (MDA-MB-231)	[102]
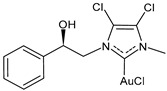	**(*S*)-7**	IC_50_ = 10.0 ± 0.5 μM (MCF-7) IC_50_ = 11.5± 0.4 μM (MDA-MB-231)	[102]
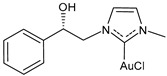	**(*R*)-8**	IC_50_ = 16.0 ± 0.8 μM (MCF-7) IC_50_ = 15.3 ± 4.5 μM (MDA-MB-231)	[102]
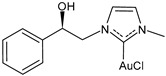	**(*S*)-8**	IC_50_ = 14.6 ± 1.0 μM (MCF-7) IC_50_ = 22.6 ± 0.4 μM (MDA-MB-231)	[102]
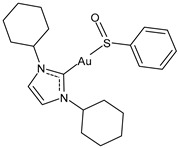	**9**	IC_50_ = 2.7 ± 0.2 μM (OVCAR-8)	[103]
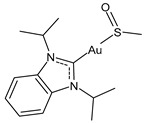	**10**	IC_50_ = 2.9 ± 0.5 μM (OVCAR-8)	[103]
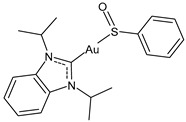	**11**	IC_50_ = 2.9 ± 0.5 μM (OVCAR-8)	[103]
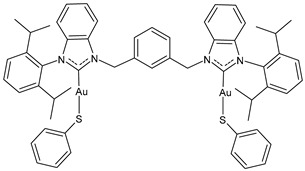	**12**	IC_50_ = 0.4 ± 0.2 μM (A2780) IC_50_ = 2.4 ± 0.2 μM (A2780cis) IC_50_ = 4 ± 0.1 μM (HCT116) IC_50_ = 7.7 ± 0.1 μM (A549) IC_50_ = 4.5 ± 0.3 μM (MDA-MB-231)	[105]
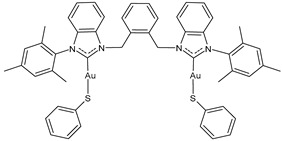	**13**	IC_50_ = 0.3 ± 0.1 μM (A2780) IC_50_ = 1.0 ± 0.3 μM (A2780cis) IC_50_ = 4.4 ± 0.2 μM (HCT116) IC_50_ = 4.4 ± 0.2 μM (A549) IC_50_ = 4.4 ± 0.2 μM (MDA-MB-231)	[105]
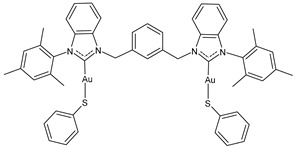	**14**	IC_50_ = 0.12 ± 0.03 μM (A2780) IC_50_ = 0.11 ± 0.01 μM (A2780cis) IC_50_ = 1.0 ± 0.1 μM (HCT116) IC_50_ = 5.8 ± 0.2 μM (A549) IC_50_ = 1.2 ± 0.5 μM (MDA-MB-231)	[105]
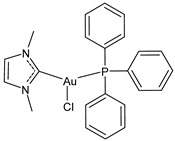	**15**	IC_50_ = 11 μM (human NSCLC with KRAS mutations) IC_50_ = 3 μM (human NSCLC without KRAS mutations)	[106]
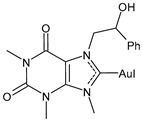	**16**	IC_50_ = 14.7 ± 1.1 μM (MDA-MB-231) IC_50_ = 73.8 ± 1.1 μM (MCF-7)	[107]

IC_50_ = half-maximal (50%) inhibitory concentration.

### 3.2. Gold–NHC Complexes as Antiviral Agents

There is a growing interest in studying other biological activities of gold–NHC complexes, including the antiviral ones. Gil-Moles et al. (2020) [34] studied some gold organometallics as inhibitors of two targets of coronaviruses SARS-CoV and SARS-CoV-2 (Table 2). In particular, the authors analyzed the compounds’ interference with the viral spike (S) protein and its interaction with the ACE2 receptor, which is essential for virus entry, as well as with the papain-like protease PL^pro^, a key enzyme in viral replication. Interestingly, the NHC complex **17** was more active than auranofin in the inhibition of spike–ACE2 interaction and against SARS-CoV-1 PL^pro^ and slightly less active than auranofin against SARS-CoV-2. Then, in 2021, the same research group [33] reported other small molecules complexed with gold(I/III) for their antiviral activity through the inhibition of SARS-CoV-1 and 2 PL^pro^. Gold(I)–NHC complexes **18–20** showed good activity, with IC_50_ values in the submicromolar range against both enzymes. Complex **19** showed antiviral activity also in a cellular model of SARS-CoV-2 infection.

Aires et al. (2022) [108] studied four gold(I) complexes (specifically two of them were triphenylphosphine and two were bimesityl–NHC derivatives) promoting antiviral effects against CHIKV infection. The triphenylphosphine complexes blocked viral replication until 99%, while the two NHC complexes, **21** and **22**, reached 50% of inhibition, without a toxic effect on cells. The higher antiviral activity of the triphenylphosphine compounds has been correlated to their higher lipophilicity and ability to cross the cell membrane. To further investigate the mechanisms of action of these two compounds, evaluating the stages of the viral replication cycle they interfere with, the authors performed time of drug addition assays; both compounds promoted a cell protective effect against infection and to inhibit the early but especially the post-entry step of the viral life cycle. These results suggested that the complexes interfere with cellular pathways and/or inhibit viral biomolecules in the cytoplasm. A significant interaction of the compounds with dsRNA was also measured, suggesting that they could inhibit viral replication by dsRNA binding.

Oliveira et al. (2024) [109] described additional studies on complex **21** and, in addition, the synthesis and antiviral activity evaluation of four other gold(I) NHC complexes. The antiviral activity was evaluated against three viruses: two arboviruses, one of Togaviridae, MAYV expressing the nanoluciferase gene reporter, and the other from the Flaviviridae, ZIKVpe243, as well as a pseudotyped vesicular stomatitis virus (VSV) expressing eGFP as an infection marker, in which the glycoprotein gene (G) was replaced by the spike protein (S) of SARS-CoV-2 (VSV-eGFP-SARS-CoV-2-S) [110]. The effect of the complex **21** and other four complexes (**23**–**26**) that are arylthiol derivatives, on virus replication, was evaluated by the infection of Vero E6 cells with the viruses described above, followed by treatment with compounds at one concentration, determined through a cell viability assay, that was 2 µM for 24–27 complexes and 10 µM for complex **21**. Regarding ZIKV, complexes **23**–**26**, and in particular **23** and **24,** showed higher antiviral activity (96% and 94% of inhibition at 2 µM, respectively), followed by **25** and **26** (88.3% and 55% of inhibition, at the same concentration 2 µM). The precursor **21** inhibited 70% of viral replication, but at a higher concentration, 10 uM. All the complexes led to a similar MAYV inhibition, ranging from 80 to 90%, with complex **26** reaching 90% of inhibition at 2 µM, the best value of the series. In general, the complexes resulted in being less active against VSV-eGFP-SARS-CoV-2-S, but with complexes **26** and **25** still achieving an inhibition of 76 and 70%, respectively. In conclusion, this study highlighted the potential antiviral activity of Au–NHC complexes and indicated complexes **23**–**26** as better antiviral candidates than complex **21**.

## 4. Conclusions

Metal-based agents form a variegate and attractive class of drugs with a number of therapeutic applications. Among these, gold complexes have gained particular attention for their biological activities. Auranofin is an FDA-approved gold-based compound for the treatment of rheumatoid arthritis. Evidence suggests that it could also be a potential therapeutic agent for different pathological conditions (inflammatory, tumoral, microbial), among which this review aimed to point out cancer and viral infections, including HIV and COVID-19. In addition, gold(I)–NHC derivatives have revealed interesting biological activities, often also superior to the standards. Most recent studies regarding auranofin and gold(I)–NHC complexes as anticancer and/or antiviral agents are summarized here. To the best of our knowledge, no or few studies have been carried out on gold(I)–NHC complexes acting both as anticancer and antiviral agents. New studies are needed to design and synthesize new gold(I)–NHC complexes with dual anticancer and antiviral activities, which could represent potent drugs for the treatment of tumors and viral infections, with promising clinical applications.

## Figures and Tables

**Table 2:** Au(I)–NHC as antiviral agents.

Structure	Compound	Antiviral Activity	Reference
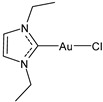	**17**	IC_50_ = 6.3 ± 1.6 μM (SARS-CoV PL^pro^) IC_50_ = 1.04 ± 0.02 μM (SARS-CoV-2 PL^pro^)	[33]
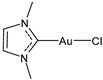	**18**	IC_50_ = 0.35 ± 0.11 μM (SARS-CoV PL^pro^) IC_50_ = 1.46 ± 0.44 μM (SARS-CoV-2 PL^pro^)	[33]
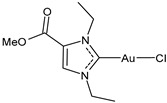	**19**	IC_50_ = 0.33 ± 0.11 μM (SARS-CoV PL^pro^) IC_50_ = 1.10 ± 0.06 μM (SARS-CoV-2 PL^pro^)	[33]
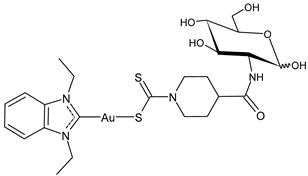	**20**	IC_50_ = 0.71 ± 0.05 μM (SARS-CoV PL^pro^) IC_50_ = 0.41 ± 0.14 μM (SARS-CoV-2 PL^pro^)	[33]
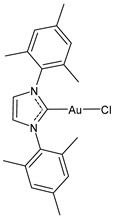	**21**	IC_50_ not given 48.5 (±17.8)% CHIKV inhibition at 10 uM 70% ZIKV inhibition at 10 uM 76.2% MAYV inhibition at 10 uM	[108,109]
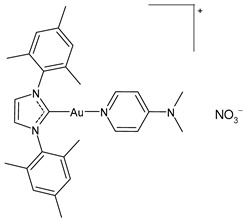	**22**	IC_50_ not given 50.7 (±15.2)% CHIKV inhibition at 10 uM	[108]
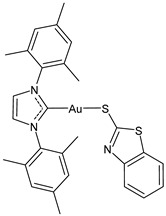	**23**	IC_50_ not given 96% ZIKV inhibition at 2 uM 80–90% MAYV inhibition at 2 uM	[109]
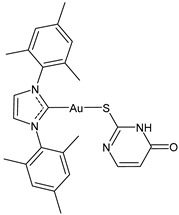	**24**	IC_50_ not given 94% ZIKV inhibition at 2 uM 80–90% MAYV inhibition at 2 uM	[109]
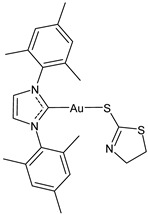	**25**	IC_50_ not given 88,3% ZIKV inhibition at 2 uM 80–90% MAYV inhibition at 2 uM	[109]
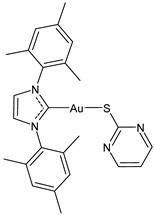	**26**	IC_50_ not given 55% ZIKV inhibition at 2 uM 90% MAYV inhibition at 2 uM	[109]
